# Anticoagulation for Prosthetic Valves

**DOI:** 10.1155/2013/346752

**Published:** 2013-11-04

**Authors:** Tsuyoshi Kaneko, Sary F. Aranki

**Affiliations:** Department of Cardiac Surgery, Brigham and Women's Hospital, 75 Francis Street, Boston, MA 02115, USA

## Abstract

Implantation of prosthetic valve requires consideration for anticoagulation. The current guideline recommends warfarin on all mechanical valves. Dabigatran is the new generation anticoagulation medication which is taken orally and does not require frequent monitoring. This drug is approved for treatment for atrial fibrillation and venous thromboembolism, but the latest large trial showed that this drug increases adverse events when used for mechanical valve anticoagulation. On-X valve is the new generation mechanical valve which is considered to require less anticoagulation due to its flow dynamics. The latest study showed that lower anticoagulation level lowers the incidence of bleeding, while the risk of thromboembolism and thrombosis remained the same. Anticoagulation poses dilemma in cases such as pregnancy and major bleeding event. During pregnancy, warfarin can be continued throughout pregnancy and switched to heparin derivative during 6–12 weeks and >36 weeks of gestation. Warfarin can be safely started after 1-2 weeks of discontinuation following major bleeding episode.

## 1. Introduction

Prosthetic valves require consideration for anticoagulation postoperatively to prevent thrombotic events. The traditional method of anticoagulation is warfarin which requires frequent blood test to check prothrombin (PT) time and International ratio (INR). American College of Cardiology and American Heart Association (ACC/AHA) have a guideline to show the adequate anticoagulation level for each position depending on the valve type: mechanical or biologic.

However, anticoagulation is not without a risk. As mentioned earlier, frequent blood testing is required and being off the target level exposes patients to risk of thrombosis and bleeding. Also, patients who are on anticoagulation have restrictions on activities to prevent bleeding events which limits lifestyle to the young patients. Warfarin carries a risk during childbearing which necessitates conversion to alternative anticoagulation method. 

In this paper, we will discuss the current guideline and show evolving new evidence which may change the way of anticoagulation with prosthetic valves. 

## 2. Current Guideline

The latest guideline from ACC/AHA in 2008 on anticoagulation for prosthesis is as follows [1].

### 2.1. Class I

1. After aortic valve replacement (AVR) with mechanical prostheses, warfarin is indicated to achieve an INR of 2.0 to 3.0. If the patient has risk factors, warfarin is indicated to achieve an INR of 2.5 to 3.5.
2. After mitral valve replacement (MVR) with mechanical valve, is indicated warfarin to achieve an INR of 2.5 to 3.5.
3. After AVR or MVR with a bioprosthesis and no risk factors, aspirin is indicated at 75 to 100 mg per day. With risk factors, warfarin is indicated to achieve an INR of 2.0 to 3.0.
4. For those patients who are unable to take warfarin, aspirin is indicated with a dose of 75 to 325 mg per day. The addition of aspirin 75 to 100 mg once daily to therapeutic warfarin is recommended for all patients with mechanical heart valves and those patients with biological valves who have risk factors.

### 2.2. Class IIa

1. During the first 3 months after AVR with a mechanical prosthesis, it is reasonable to give warfarin to achieve an INR of 2.5 to 3.5.
2. During the first 3 months after bioprosthesis, it is reasonable to give warfarin to achieve INR of 2.0 to 3.0.

### 2.3. Class IIb

1. In high-risk patients with prosthetic heart valves in whom aspirin cannot be used, it may be reasonable to give clopidogrel (75 mg per day) or warfarin to achieve an INR of 3.5 to 4.5.

## 3. Antiplatelet Drugs for Prosthetic Valves

Although current guideline shown previously recommends addition of aspirin to warfarin for mechanical valves and bioprosthetic valves with risk factors, antiplatelet drugs are not without a risk. The most recent meta-analysis from Cochrane review in 2013 has looked into this issue [2]. They included 13 studies with 4122 patients in this metaanalysis. Compared to oral anticoagulation (warfarin) alone, addition of antiplatelet agent reduced the risk of thromboembolic events (odds ratio (OR) 0.43, *P* < 0.0001) and total mortality (OR 1.57, *P* < 0.0001). Aspirin and dipyridamole had similar outcomes. However, risk of major bleeding was increased when antiplatelet agents were added (OR 1.58, *P* = 0.006). Author's recommendations are similar to the guidelines to use antiplatelet therapy for patients receiving mechanical valves and bioprosthetic valves with high risk factors such as atrial fibrillation, venous thromboembolism, left ventricular dysfunction, and hypercoagulable state. Risk of bleeding was the lowest with low dose aspirin and the benefits were similar. 

## 4. New Anticoagulants

A new generation of anticoagulation has been developed and has been tested clinically. Dabigatran etexilate is oral direct thrombin inhibitor and its safety and efficacy on atrial fibrillation [3, 4] and deep venous thrombosis [5, 6] have been reported. The benefits of this medication compared to warfarin are the following:

1. half life of 12 hours and it does not require frequent INR monitoring;
2. there are less drug interactions compared to warfarin;
3. rapid clinical onset with predictable dose response.

In a swine model, dabigatran showed similar efficacy to enoxaparin without adverse effects [7]. However, the recently published large randomized control study, namely, RE-ALIGN trial (randomized, phase II study to evaluate the safety and pharmacokinetics of oral dabigatran etexilate in patients after heart valve replacement), was terminated early due to adverse effect in the dabigatran group [8]. This trial studied two groups who underwent implantation of mechanical valve (aortic and mitral) within 7 days or within 3 months. Dabigatran was dose-adjusted according to kidney function to obtain trough plasma level of at least 50 ng per milliliter. The trial was terminated prematurely after the enrollment of 252 patients because of an excess of thromboembolic and bleeding events among patients in the dabigatran group. Following this report, Food and Drug Administration (FDA) announced a statement to contraindicate dabigatran for mechanical heart valves. At the current moment, dabigatran is contraindicated and should not be used for patients with mechanical valves [9].

## 5. New Generation Mechanical Valve

The On-X mechanical valve (Medical Carbon Research Institute, Austin, TX, USA) uses pure pyrolytic carbon and does not require silicon carbide additives to gain sufficient strength and wear resistance. It also has flared inlet design to reduce inlet turbulence and an elongated orifice to organize flow and reduce exit losses. It creates streamlined blood flow and leads to improved fluid dynamics and less blood damage than previous valve models [10]. Long-term outcomes on this valve have been reported to have low thrombosis rate, 0% in some literature [11, 12]. The carbon technology and transvalve flow patterns make this valve a low thrombogenicity and thromboembolism is uncommon even in a population in whom 40% have no or inadequate anticoagulation [13]. The On-X valve on label does not comment on any requirement for anticoagulation given these product characteristics. 

Given these results, Prospective Randomized On-X Anticoagulation Clinical Trial (PROACT) study for high risk AVR patients at 36 centers in the United States was conducted [14]. In this trial, patients were randomized to either low dose warfarin (INR 1.5–2.0) or to standard dose warfarin (INR 2.0–3.0) for three months following mechanical AVR using On-X valve. All patients received 81 mg daily aspirin. The patients in low dose warfarin had mean INR of 1.89 and standard group had INR of 2.50. The low dose group had significantly lower major and minor bleeding event rates and there was no significant difference between the two groups in terms of stroke, transient ischemic attack (TIA), or total neurological events. There was no significant difference in all-cause mortality between the low dose and standard dose groups. This report is expected to increase the use of new generation mechanical valves requiring lower INR target. 

## 6. Anticoagulation for Bioprosthesis

For bioprosthesis, current guidelines state that it is reasonable to give warfarin for the first 3 months to a target an INR of 2.0 to 3.0 as Class IIa recommendation. No anticoagulation is needed in low risk patients as Class I recommendation. However, there is a recent paper which challenged this recommendation. 

Mérie and associates reported outcomes of 4075 patients from Danish National Patient Registry who underwent AVR using bioprosthetic valves [15]. Patients not treated with warfarin compared to those treated with warfarin had higher rate of strokes, thromboembolic events, bleeding incidents, and cardiovascular deaths within 30 to 89 days after surgery and within 90 to 179 days after surgery. The cardiovascular deaths after 180 days were similar between the two groups. Following this result, authors recommended use of warfarin for 6 months following bioprosthetic implantation in aortic position. 

## 7. Anticoagulation Management during Pregnancy

One of the adverse effects of warfarin is increased fetal morbidity and mortality. The low molecular weight of this drug allows transplacental passage and causes embryopathy. The most critical period for intrauterine exposure to warfarin is considered to be 6–9 weeks of gestation, which is organogenesis period. Risk of embryopathy may be <10% in the first trimester and dramatically reduced to a level similar to that of an untreated population after second trimester [16]. The incidence is also known to be dose dependent; decreased adverse event is seen when warfarin <5 mg is used rather than >5 mg [17]. The risk of embryopathy can be decreased by switching to unfractionated or low-molecular weight heparin since these drugs do not cross the placental barrier. However, use of heparin is associated with increased risk of maternal thromboembolic events, which is reported to be up to 12–24% [1].

The current guideline from ACC/AHA recommends use of anticoagulation throughout pregnancy. Class I recommendation is assigned to the use of continuous intravenous heparin, subcutaneous heparin or low-molecular-weight heparin, during weeks 6–12 of gestation in patients who wish to discontinue warfarin during this period or during pregnancy [1]. General consensus between physicians is that at week 36 of gestation, warfarin should be switched to heparin prior to delivery. The guideline added that there is paucity of data on the efficacy of anticoagulation regimen during pregnancy. 

Warfarin is associated with greater risk of fetal mortality compared to heparin, but this is not observed when administered in low dose. Heparin is less effective as an anticoagulant to prevent thromboembolism. Replacement of warfarin to heparin during the first trimester is shown to reduce the risks [18]. It is important to inform these risks to the patients and decide the appropriate anticoagulation regimen. 

## 8. Anticoagulation Management in Case of Severe Systemic Bleeding

Dilemma for mechanical valves occurs when the patients experience major bleeding episode. Balancing the benefit of anticoagulation (prevention of valve thrombosis) and the risk (worsening of the bleeding) makes this problem difficult. 

The question of when to restart anticoagulation is a difficult one. Phan and associates reported 52 patients following valve prosthesis implantation and intracranial hemorrhage and showed that discontinuation of anticoagulation for 1-2 weeks has a low probability of thromboembolic events in patients with high embolic risk [19]. There has been, report of discontinuation of anticoagulation for 3 months without thromboembolic or thrombosis events [20]. 

These data show that for some patients with major bleeding episode on anticoagulation, it can be safely held for 7–14 days with low probability of thromboembolic phenomenon. Most importantly, in major bleeding cases, we must weigh the risk of bleeding and valve thrombosis and adjust the period of discontinuing anticoagulation based on individual case. 

## 9. Anticoagulation Management for Surgical Procedures

For patients who are getting minor surgeries such as dental work, cataract surgery, or cutaneous surgery, warfarin needs to be discontinued or kept below the low end of the therapeutic range (e.g., INR 1.7 to 2.3) [21]. For dental procedures, tranexamic acid mouthwash can be used to limit the bleeding. For cutaneous surgery or cataract, the minimal bleeding allows the surgeons to continue anticoagulation. 

For high risk surgeries such as hip replacement or cancer surgery, bridging anticoagulation should be given. Warfarin is stopped 5,6 days prior to surgery with goal to achieve INR < 1.5 at the time of operation and bridging anticoagulation using unfractionated heparin or low molecular weight heparin until the day of surgery. Warfarin is started after 24 hrs of surgery following confirmation of hemostasis and warfarin is started at the same time. Heparin derivative is continued until INR is therapeutic. There is no large study justifying this strategy and currently large randomized control study (BRIDGE study which is National Institutes of Health—sponsored randomized Trial, unique identifier NCT00786474) is being conducted which aims to determine whether bridging anticoagulation is needed in patients with atrial fibrillation who are receiving warfarin and need to undergo surgery/procedure.

## 10. Conclusion

Valve implantation requires consideration for anticoagulation postoperatively. A new generation of valves is currently being tested to decrease the bleeding events without increasing the risk of thromboembolism and valve thrombosis. There are cases such as pregnancy or major bleeding which require augmentation of anticoagulation. The anticoagulation regimen maybe tailored to each individual case considering the risk and the benefit. It is important for physicians to understand the risks and to discuss these risks with the patients. 
